# Barriers and facilitators of childhood immunization: sociodemographic and knowledge-related determinants among parents in rural and urban Khyber Pakhtunkhwa, Pakistan

**DOI:** 10.1017/S0950268826101277

**Published:** 2026-03-23

**Authors:** Jannat Ashfaq, Kifayat Ullah, Farhad Ali Khattak, Muhammad Hakim, Saima Afaq, Khalid Rehman, Asif Bettani, Zia ul Haq

**Affiliations:** 1 Fauji Foundation Hospital, Kohat, Pakistan; 2 Federal Directorate of Immunization, Islamabad, Pakistan, Pakistan; 3 Institute of Public Health & Social Sciences, Khyber Medical University, Pakistan; 4 Department of Health Sciences, University of York, UK; 5 UKHSA, UK

**Keywords:** Childhood immunization, vaccination defaulters, immunization barriers, maternal education, Vaccine refusal

## Abstract

Immunization is critical for reducing vaccine-preventable disease morbidity and mortality, yet coverage disparities persist in low-resource settings. This mixed-methods study describes characteristics of childhood immunization defaulters and explores barriers to vaccine adherence in Khyber Pakhtunkhwa, Pakistan. We recruited 380 caregivers from three tehsils in District Kohat of Khyber Pakhtunkhwa from February to July 2023, whose children under 2 years had not completed the Expanded Program on Immunization (EPI)-recommended schedule. Quantitative data from validated questionnaires and immunization cards underwent descriptive and regression analyses; qualitative interviews explored non-adherence reasons. Most respondents were fathers (96.05%); 41.84% resided in rural areas. Initial coverage was high for BCG (97.89%) and OPV0 (100%) but declined for Penta3 (26.05%) and Measles2 (4.21%). Most children (73.95%) were under 4 months. Rural defaulters were more prevalent than urban (41.84% vs. 34.47%, p < 0.001), and 89.47% had mothers with ≤high school education. While 95.26% had heard of vaccines, only 49.47% knew the EPI starting age. Defaulters with higher knowledge progressed further through the schedule (AOR: 4.55, p = 0.05). Qualitative themes included poor healthcare access, cultural norms, religious misconceptions, and migration disruptions. Interventions addressing maternal education, rural access, and knowledge gaps are essential to reduce immunization default.

## Key results

- About 73.95% of defaulters are infants under 4 months, indicating failure to retain families during the most intensive vaccination period when multiple clinic visits are required.
- Despite 95.26% having heard of vaccines, only 49.47% knew when to start EPI, and 39.74% could name four vaccine-preventable diseases, revealing superficial awareness without actionable knowledge.
- Rural residence, low maternal education, and limited healthcare access cluster together 88% of children who defaulted on later vaccines (MR2) were rural residents with poorly educated mothers living >5 km from facilities.

## Introduction

Childhood vaccination is a vital public health measure that significantly reduces the burden of vaccine-preventable diseases (VPDs) and improves child survival rates [1]. When children receive all recommended doses on time, the risk of VPDs decreases considerably. However, many caregivers fail to complete the vaccination schedule, resulting in a large number of children becoming defaulters, defined as those who do not receive all recommended vaccinations before their first birthday [2]. In low- and middle-income countries (LMICs), VPDs account for more than 3 million deaths among young children each year [3]. Owing to their low cost and long-term health benefits, vaccination programmes such as the Expanded Program on Immunization (EPI) remain a cornerstone of global public health strategies [4]. Despite global efforts, vaccination coverage has declined in recent years. Worldwide, immunization rates among children aged 0–23 months fell from 86% in 2019 to 81% in 2021, the lowest in more than a decade, with the majority of unvaccinated children residing in LMICs [5]. Alarmingly, 58% of children at risk of VPDs have not received a single dose [6].

In Pakistan, the EPI was launched in 1978 with support from the World Health Organization (WHO), UNICEF, and other partners. The programme currently provides children under 12 months with vaccines against 12 VPDs, including tuberculosis, diphtheria, tetanus, pertussis, poliomyelitis, hepatitis B, *Haemophilus influenzae* type b, pneumonia, meningitis, typhoid, rubella, measles, and rotavirus diarrhoea [7]. Despite this, coverage remains suboptimal. As of 2023, nearly 400,000 children in Pakistan had not received their first dose of the pentavalent (Penta-1) vaccine [8]. According to the National Demographic and Health Survey, only 66% of children aged 12–23 months are fully immunized, highlighting persistent and significant gaps in national coverage [9].

Although the benefits of immunization are well established, defaulting remains a major barrier to achieving optimal vaccination rates [10]. Incomplete vaccination has been associated with multiple interrelated factors, including low socioeconomic status [11], limited parental knowledge and negative attitudes [12], poor accessibility of health services [13], parental employment constraints [14], cultural and religious beliefs [15], and significant geographical disparities [16]. Consequently, Pakistan’s vaccination rates continue to lag behind those of many neighbouring countries [17]. Critical evidence gaps persist regarding additional barriers, such as transportation challenges [18], perceptions of vaccine importance [19], weaknesses in the vaccine supply chain and health infrastructure [20], and the specific roles of healthcare professionals and maternal behaviours in vaccination adherence [21]. A better understanding of these multifaceted barriers is essential for designing context-specific interventions and strengthening immunization programmes in resource-limited settings. However, limited evidence exists regarding the specific characteristics of vaccine defaulters and factors associated with their progression through immunization schedules. Understanding the profile of defaulters, their knowledge levels, and the contextual barriers they face can inform targeted intervention strategies. This study, therefore, aims to determine the sociodemographic and knowledge-related characteristics of childhood immunization defaulters between parental knowledge levels and vaccination coverage patterns and explore barriers and facilitators to vaccine adherence among caregivers of children under 2 years in rural and urban areas of Khyber Pakhtunkhwa, Pakistan.

## Methodology

A mixed-method study was conducted in Kohat District, Khyber Pakhtunkhwa, Pakistan, from February to July 2023. The study adopted a sequential explanatory design consisting of two phases. The quantitative phase (cross-sectional) identified sociodemographic and knowledge-related determinants of childhood immunization default, while the qualitative phase, using a phenomenological approach, explored the underlying reasons for non-adherence. This mixed-method design allowed for an in-depth understanding of both measurable and contextual factors influencing immunization practices.

### Ethical considerations

Ethical approval was obtained from the Institutional Review Board of Khyber Medical University, Peshawar (KMU/IPHSS/Ethics/2022/SO/0182), and administrative permission was granted by the District Health Authority of Kohat. Written and oral informed consent were obtained from all participants prior to enrolment. Participants were informed about the study’s purpose, confidentiality of information, voluntary participation, and their right to withdraw at any stage without penalty.

### Study population and setting

The study targeted caregivers of children under 2 years of age who had not completed the full immunization schedule under Pakistan’s EPI. An *immunization defaulter* was defined as a child who had reached the recommended age for one or more vaccine doses according to the national EPI schedule but had not received those age-appropriate doses at the time of the survey, and no additional grace period beyond the scheduled age was applied in determining missed doses.

*Parental defaulting* refers to the caregiver’s failure to ensure the timely completion of the EPI vaccination schedule for their child. Caregivers employed in healthcare delivery or residing in the study area for less than 12 months and children with severe illness were excluded to reduce bias and ensure comparable exposure to local health services.

The study was carried out in Kohat District, which comprises four administrative tehsils: Kohat, Lachi, Gumbat, and Dara Adam Khel. Three tehsils, Kohat, Lachi, and Gumbat, were randomly selected. Each selected tehsil was further stratified into urban and rural zones based on official administrative classifications, considering the availability of essential services. The district’s healthcare network includes district and tehsil headquarters hospitals, rural health centres (RHCs), and basic health units (BHUs), all providing routine immunization under the EPI framework.

### Sampling technique

A multistage cluster sampling approach was used. In the first stage, three of the four tehsils were selected randomly. In the second stage, urban and rural strata within each tehsil were selected randomly. In the third stage, EPI centres were chosen randomly from these strata until the target sample size was reached, resulting in 41 EPI centres. In the qualitative phase, a purposive subsample of caregivers from the quantitative phase was selected. Only participants who had consented to follow-up interviews were approached for in-depth discussions.

### Sample size

The sample size was calculated using the WHO sample size calculator, assuming 66% (32) immunization coverage (Pakistan Demographic and Health Survey 2017–2018), a 5% margin of error, and a 95% confidence level. The required sample size was 380 caregivers. Additionally, 16 caregivers were included for semi-structured qualitative interviews. To account for potential non-response and incomplete data, the recruitment target was inflated. Assuming an expected response rate of approximately 90% (non-response ≈10%), the number of caregivers to approach was estimated as 380 ÷ 0.90 ≈ 422. For operational feasibility, 420 caregivers were approached to ensure at least 380 completed responses.

### Data collection

Demographic details of defaulter children were obtained from selected EPI centres. Caregivers were then contacted via home visits. Informed consent was reconfirmed at each visit before data collection. Quantitative data were collected through face-to-face structured interviews using a validated questionnaire adapted from Cao et al. [22], which was adopted for the local context by pilot testing. The questionnaire was administered in Pashto and Urdu, the predominant local languages, by trained interviewers fluent in both. It covered demographic characteristics of caregivers and children, healthcare provider details, and immunization records. Vaccination status was verified using immunization cards, where available; in their absence, caregiver recall and hospital records were used. For the qualitative phase, data were collected through semi-structured interviews using an interview guide designed to capture barriers to immunization adherence. Interviews were conducted at participants’ homes or EPI centres, depending on participant preference. Each interview was audio-recorded, with field notes taken simultaneously. Two trained interviewers (one male, one female) with backgrounds in public health conducted the interviews, ensuring cultural sensitivity and minimizing interviewer bias. A pilot study with 20 participants was conducted prior to data collection to test the feasibility, clarity, and flow of instruments. Based on feedback, minor modifications were made to the wording and order of questions.

### Data analysis

#### Quantitative phase

We used R (RStudio) for our data analysis. Statistical significance was set at *p* ≤ 0.05.

#### Variables and covariates

Caregiver knowledge regarding immunization was assessed using seven items covering VPDs, immunization schedules, and potential side effects. Each correct response was assigned a score of 1, resulting in a composite knowledge score ranging from 0 to 7. For the primary analysis, this score was dichotomized to define the binary outcome variable *Adequate Vaccination Knowledge.* A predefined cutoff of ≥5 correct responses (≥71%) was used to classify participants as having adequate knowledge (coded as 1) versus inadequate knowledge (coded as 0). This threshold was selected to reflect a substantial majority of correct responses, indicating a functional level of understanding relevant for vaccination decision-making. Caregiver age was categorized into three equal intervals to ensure balanced group comparisons. The quantitative analysis focused on two main outcomes: (1) vaccination coverage, measured as receipt of age-appropriate vaccine doses (OPV1/ROTA1/PENTA1; OPV2/ROTA2/PENTA2; OPV3/IPV1/PENTA3; MR1; MR2) among target children; and (2) caregiver knowledge related to immunization. Associations between caregiver knowledge and vaccination uptake were initially examined using chi-square tests. Binary logistic regression was used to estimate crude associations, followed by multivariable logistic regression to obtain adjusted estimates, with caregiver knowledge specified as the dependent variable in the final model. Covariate selection for the adjusted model followed a prespecified, theory-informed approach to minimize data-driven bias. Covariates were selected a priori based on their established role as potential confounders in the immunization literature, evidence of association in bivariate analyses, and adequate cell counts in contingency tables to ensure model stability. The final model reports adjusted odds ratios (aORs) with 95% confidence intervals for the main exposures of interest.

#### Qualitative phase

A qualitative phase was conducted to complement the quantitative findings. A total of 16 semi-structured interviews were carried out in the local language (Pashto) at participants’ homes. The interviews were facilitated by a trained public health professional from the same district who had received additional training in qualitative interviewing techniques specific to this study. Interviews were conducted until thematic saturation was reached, defined as the point at which no new codes or insights emerged from the data. The process was iterative: after every few interviews, transcripts and field notes were reviewed and discussed by the research team to identify emerging patterns and assess whether additional interviews were likely to yield new information. Saturation was determined when two consecutive interviews produced no novel codes or themes. A senior qualitative researcher oversaw this process through continuous review of transcripts and memos to ensure analytical depth and completeness.

All interviews were audio-recorded with participant consent, transcribed verbatim in Pashto, and translated into English where required. The data were analysed using Braun and Clarke’s six-step thematic analysis framework: familiarization, coding, searching for themes, reviewing themes, defining themes, and writing up. Two researchers independently coded transcripts to enhance reliability, and discrepancies were resolved through discussion. Codes were grouped into categories and synthesized into broader themes, which were validated against the raw data to ensure accuracy. NVivo 12 software was used to manage and organize the analysis. To enhance rigor, intercoder agreement was checked on a subset of transcripts, and direct participant quotations were used to illustrate key themes.

## Results

### Quantitative part

A total of 380 caregivers of children under 2 years of age participated in the study. The majority resided in Tehsil Kohat (59.21%), followed by Tehsil Lachi (21.05%) and Tehsil Gumbat (19.74%). Fathers comprised 96.05% of respondents, while mothers and grandparents made up only 1.58% and 2.37%, respectively. Male children represented 60.53% of the sample, and most children (68.69%) were aged 3–4 months. Fathers had higher levels of education, with 43.95% have a college degree or higher, compared to only 10.53% of mothers.

Rural residency was more prevalent (41.84%) compared to urban (34.47%) and urban–rural fringe areas (23.68%). Immunization coverage for BCG and OPV0 was high, at 97.89% and 100%, respectively, but declined for subsequent vaccines, Penta1 at 95%, falling to 26.05% for Penta3. Measles vaccination coverage was particularly low, with Measles1 at 21.84% and Measles 2 at 4.21%. Overall, 73.68% of children defaulted on multiple vaccines, particularly those under 4 months old (73.95%). Accessibility was a major issue, with 60.79% living more than 5 km from a healthcare facility and 62.37% reporting travel times of over 20 min. Children of illiterate mothers were 2.4 times more likely to default on vaccinations. While general awareness of vaccination was high (95.26%), only 49.47% of respondents knew the correct starting age for EPI services, and just 39.74% could identify four VPDs. Health workers were the primary source of immunization information (59.74%), followed by television (17.11%). Most respondents (97.89%) possessed immunization cards, indicating strong documentation practices. (Supplementary Table S1).

**Figure 1.**
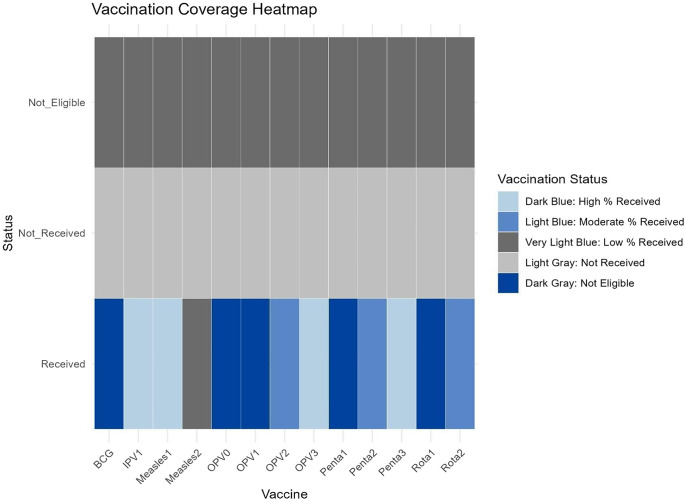
Heatmap’s gradient coverage across different vaccine types and stages. BCG: bacillus Calmette–Guérin vaccine; Measles1 / Measles2: measles-rubella vaccine first and second doses; OPV0 / OPV1 / OPV2: oral polio vaccine at birth, 6 weeks, and 10 weeks respectively; Polio1: inactivated polio vaccine (IPV); PENTA1 / PENTA2 / PENTA3: pentavalent vaccine (DTP-HepB-Hib) at 6, 10, and 14 weeks; ROTA1 / ROTA2: rotavirus vaccine first and second doses.

The heatmap Plot (Figure 1) visualizes vaccination status across different vaccine types and stages, categorized as ‘Received’, ‘Not Received’, and ‘Not Eligible’, with percentages represented by colour intensity. The darker shades of blue indicate higher percentages, reflecting better vaccination coverage. Notably, vaccines such as BCG and OPV-0 have the highest coverage, as evidenced by the dominance of deep blue in the ‘Received’ category. Conversely, vaccines like Measles 2 and Rotavirus 2 show relatively lower coverage, with lighter blue shades in the ‘Received’ category and more representation in the ‘Not Received’ category. The ‘Not Eligible’ category, seen for some vaccines, likely represents infants who had not yet reached the age for those vaccine doses at the time of data collection. This is particularly evident for vaccines requiring multiple doses or those scheduled for later stages, such as Measles 2 and Rotavirus 2.

Our study demonstrates significant associations between socioeconomic and geographic factors and immunization defaulting across key vaccine types (Supplementary Table S2). Maternal education was significantly associated with vaccine uptake (p = 0.014). Among children defaulting on MR2, 69% (11/16) had mothers with only primary education, while just 6.3% (1/16) had secondary education, and none had college-level education. Paternal education also showed a strong relationship (p = 0.001). Among MR2 defaulters, 69% (11/16) had fathers with only secondary education, while only 19% (3/16) had college and above education. Just 13% (2/16) had a high school education, and none were illiterate. Residency significantly influenced immunization status (p < 0.001). Among MR2 defaulters, 88% (14/16) belonged to rural areas, whereas only 6.3% (1/16) each were from urban and fringe urban-rural settings. Distance from healthcare facilities was nearly significant (p = 0.052). Among MR2 defaulters, 38% (6/16) lived more than 10 km away from a health facility, 63% (10/16) lived 5–10 km away, while none lived within 5 km. Availability of immunization cards was also a significant factor (p < 0.001). Among MR2 defaulters, only 6.3% (1/16) lacked an immunization card, whereas 94% (15/16) had one. Geographic terrain was significantly associated with immunization status (p = 0.019). Among MR2 defaulters, 69% (11/16) were from plain areas, 19% (3/16) from hilly regions, and 13% (2/16) from mountainous areas.

Antigen-specific coverage revealed variation in default rates across different vaccines. The highest completion rates were observed for BCG at 89.1%, followed by DPT (3 doses) at 74.5%, Polio (3 doses) at 72.3%, and Hepatitis B (3 doses) at 71.2%. In contrast, the measles vaccine (first dose) showed the lowest coverage at 67.4%, indicating a notable drop-off in immunization adherence as the schedule progresses.

Table 1 highlights the relationship between caregivers’ knowledge about (low vs. high) vaccination coverage across various characteristics of children, caregivers, and healthcare providers. Among child characteristics, children aged over 12 months were more likely to have caregivers with high knowledge (75%, p = 0.025), while gender showed no significant association (p = 0.43). Regarding caregiver characteristics, rural caregivers were more likely to have high knowledge (44%, p = 0.026), and fathers with college-level education or higher were significantly associated with high knowledge (53%, p = 0.005). Healthcare provider factors, such as distance to facilities (p = 0.014) and travel time (p = 0.020), were also significant. Longer distances and travel times were linked to caregivers with high knowledge. Additionally, children in the low-knowledge group were more likely to default on early-stage vaccines like OPV1, Rota1, and Penta1 (70%, p = 0.044), whereas adherence to later-stage vaccines was better in the high-knowledge group (75%).

**Table 1.** Characteristics of respondents by level of vaccination knowledge (low vs. high)

Characteristic	Low knowledge N = 183^1^	High knowledge N = 197^1^	*p*-value^2^
*Children characteristics*			
Age in months			
Less than 4 months	146 / 281 (52%)	135 / 281 (48%)	0.025
4–8 months	0 / 0 (NA%)	0 / 0 (NA%)	
8.1–12 months	33 / 83 (40%)	50 / 83 (60%)	
More than 12 months	4 / 16 (25%)	12 / 16 (75%)	
Gender			0.43
Male	115 / 230 (50%)	115 / 230 (50%)	
Female	68 / 150 (45%)	82 / 150 (55%)	
*Respondent characteristics*			
Terrain			
Mountain	2 / 8 (25%)	6 / 8 (75%)	0.27
Hill	9 / 23 (39%)	14 / 23 (61%)	
Plain	172 / 349 (49%)	177 / 349 (51%)	
Residency			0.026
Urban	75 / 183 (41%)	56 / 197 (28%)	
Urban Rural fringe area	36 / 183 (20%)	54 / 197 (27%)	
Rural	72 / 183 (39%)	87 / 197 (44%)	
Maternal education			0.071
Illiterate	7 / 13 (54%)	6 / 13 (46%)	
Primary	36 / 98 (37%)	62 / 98 (63%)	
secondary	26 / 48 (54%)	22 / 48 (46%)	
High School	97 / 181 (54%)	84 / 181 (46%)	
College & Above	17 / 40 (43%)	23 / 40 (58%)	
Paternal education			0.005
Illiterate	3 / 3 (100%)	0 / 3 (0%)	
Primary	8 / 9 (89%)	1 / 9 (11%)	
secondary	34 / 91 (37%)	57 / 91 (63%)	
High school	59 / 110 (54%)	51 / 110 (46%)	
College & above	79 / 167 (47%)	88 / 167 (53%)	
Use of mobile			>0.99
No	7 / 15 (47%)	8 / 15 (53%)	
Yes	176 / 365 (48%)	189 / 365 (52%)	
*Children characteristics*			
Ethnicity			0.97
Muslim	182 / 379 (48%)	197 / 379 (52%)	
Non-Muslim	1 / 1 (100%)	0 / 1 (0%)	
Birth place			0.70
Hospital	109 / 218 (50%)	109 / 218 (50%)	
Health centre	70 / 153 (46%)	83 / 153 (54%)	
Home	4 / 9 (44%)	5 / 9 (56%)	
Resident status			0.91
Permanent resident	172 / 359 (48%)	187 / 359 (52%)	
Migrant from another city	3 / 6 (50%)	3 / 6 (50%)	
Migrant from another province	8 / 15 (53%)	7 / 15 (47%)	
Healthcare provider characteristics			
Immunization provider			0.31
DHQ	92 / 175 (53%)	83 / 175 (47%)	
BHU/RHC	52 / 121 (43%)	69 / 121 (57%)	
Civil dispensary	33 / 74 (45%)	41 / 74 (55%)	
Administration of vaccines at home	6 / 10 (60%)	4 / 10 (40%)	
Distance from home			0.014
<5 km	48 / 79 (61%)	31 / 79 (39%)	
>5 km–10 km	109 / 231 (47%)	122 / 231 (53%)	
>10 km	26 / 70 (37%)	44 / 70 (63%)	
Traveling time			0.020
<20 min	47 / 76 (62%)	29 / 76 (38%)	
20–40 min	109 / 237 (46%)	128 / 237 (54%)	
>40 min	27 / 67 (40%)	40 / 67 (60%)	
Availability of immunization book			>0.99
No	4 / 8 (50%)	4 / 8 (50%)	
Yes	179 / 372 (48%)	193 / 372 (52%)	
Defaulter for group of vaccine types			0.044
OPV1, ROTA1 & PENTA1	14 / 20 (70%)	6 / 20 (30%)	
OPV2, ROTA2, PENTA 2	64 / 126 (51%)	62 / 126 (49%)	
OPV3, IPV 1 & Penta3	67 / 134 (50%)	67 / 134 (50%)	
MR1	34 / 84 (40%)	50 / 84 (60%)	
MR2	4 / 16 (25%)	12 / 16 (75%)	

1 n / N (%).

2 Pearson’s Chi-squared test/Fisher’s exact test: BHU: basic health unit; DHQ: district headquarter hospital; EPI = Expanded Programme on Immunization; IPV: Inactivated Polio Vaccine; MR: measles-rubella vaccine; OPV: oral polio vaccine; PENTA: pentavalent vaccine (DTP-HepB-Hib); RHC: rural health centre; ROTA: rotavirus vaccine; VPD: vaccine-preventable disease.

Univariate and multivariate logistic regression analyses were conducted (Table 2) to evaluate factors associated with respondents’ knowledge about vaccination. Maternal education did not show a statistically significant association with knowledge in either univariate or multivariate analysis. For instance, respondents with college-level education and above had an odds ratio (OR) of 1.85 (95% CI: 0.49, 7.20; p = 0.4) in the multivariate model compared to those who were illiterate. Resident status of the child also did not significantly influence knowledge, with migrants from another city showing an OR of 1.41 (95% CI: 0.24, 8.72; p = 0.7) and migrants from another province an OR of 0.83 (95% CI: 0.27, 2.46; p = 0.7) compared to permanent residents in the multivariate model. Travel time to healthcare facilities, however, demonstrated a stronger relationship with vaccination knowledge. Respondents traveling more than 40 min to a healthcare facility were more likely to have knowledge about vaccination, with an OR of 1.97 (95% CI: 0.96, 4.08; p = 0.06) compared to those traveling less than 20 min, although this did not reach statistical significance in the multivariate model. Importantly, respondents who had heard about vaccination were nearly three times as likely to have vaccination knowledge (OR: 2.84; 95% CI: 0.98, 9.48; p = 0.06) compared to those who had not, with a borderline significant p-value. Finally, defaulter status for specific antigen groups showed varying levels of association. Respondents whose children defaulted on MR2 were significantly more likely to have vaccination knowledge, with an OR of 4.55 (95% CI: 1.04, 23.5; p = 0.05) in the multivariate model.

**Table 2.** Determinants of adequate vaccination knowledge using unadjusted and adjusted odds ratios from logistic regression models

Characteristic	Univariate			Multivariate		
	OR^1^	95% CI^1^	p- value	AOR^1^	95% CI^1^	*p*-value
Maternal education						
Illiterate	–	–		–	–	
Primary	2.01	0.62, 6.69	0.2	1.99	0.58, 6.95	0.3
secondary	0.99	0.29, 3.48	>0.9	1.09	0.30, 4.10	0.9
High school	1.01	0.32, 3.25	>0.9	1.28	0.38, 4.45	0.7
College & above	1.58	0.45, 5.74	0.5	1.85	0.49, 7.20	0.4
Resident status child characteristics						
Permanent resident	–	–		–	–	
Migrant from another city	0.92	0.17, 5.03	>0.9	1.41	0.24, 8.72	0.7
Migrant from another province	0.80	0.28, 2.29	0.7	0.83	0.27, 2.46	0.7
Traveling time to healthcare provider						
<20 min	–	–		–	–	
20–40 min	1.90	1.13, 3.26	0.017	1.67	0.97, 2.90	0.06
>40 min	2.40	1.23, 4.75	0.011	1.97	0.96, 4.08	0.06
Have you ever heard about vaccination						
No	–	–		–	–	
Yes	2.94	1.08, 9.31	0.045	2.84	0.98, 9.48	0.06
Defaulter for group of types						
OPV1, ROTA1 & PENTA1	–	–		–	–	
OPV2, ROTA2, PENTA 2	2.26	0.85, 6.73	0.12	1.96	0.70, 6.04	0.20
OPV3, IPV 1 & Penta3	2.33	0.88, 6.92	0.10	1.88	0.66, 5.86	0.20
MR1	3.43	1.25, 10.5	0.02	2.79	0.95, 8.96	0.06
MR2	7.00	1.70, 34.4	0.01	4.55	1.04, 23.5	0.05

1 CI: confidence interval; IPV: inactivated polio vaccine; MR: measles-rubella vaccine; OPV: oral polio vaccine; OR: odds ratio; PENTA: pentavalent vaccine (DTP-HepB-Hib); ROTA: rotavirus vaccine.

### Qualitative part

Following the quantitative survey, a qualitative component was undertaken to explore the underlying reasons and contextual narratives behind immunization behaviours. We conducted semi-structured interviews with 16 caregivers. Table 3 summarizes the demographic profile of these participants, illustrating a mix of genders, ages, residential backgrounds (urban/rural), and familial roles. Thematic analysis of these interviews yielded rich insights, with four major themes emerging:

**Table 3.** Demographic characteristics of the interview participants for qualitative data

S.No.	Identification code	Gender	Age in years	Relation with defaulter child	Urban / Rural
1	PR – 1	Male	37	Father	Urban
2	PR – 2	Male	53	Father	Urban
3	PR – 3	Male	65	Grand father	Rural
4	PR – 4	Female	46	Mother	Urban
5	PR – 5	Male	41	Father	Urban
6	PR – 6	Male	32	Father	Urban
7	PR – 7	Male	65	Grand father	Rural
8	PR – 8	Female	37	Mother	Urban
9	PR – 9	Male	35	Father	Urban
10	PR – 10	Female	21	Mother	Urban
11	PR – 11	Male	30	Father	Urban
12	PR – 12	Male	59	Grand father	Urban
13	PR – 13	Male	31	Father	Urban
14	PR – 14	Male	70	Grand father	Rural
15	PR – 15	Male	35	Father	Urban
16	PR – 16	Female	55	Mother	Rural

#### Accessibility challenges

In summarizing the qualitative data, a prominent theme emerged regarding challenges associated with accessing vaccination centres. Communities residing in remote areas faced difficulties reaching the centres at the scheduled vaccination times for their children. Participants cited various obstacles related to accessibility, including long distances between their homes and the centres, insufficient transportation options, and difficult geographical terrain.

*Going to the vaccination centre along with a small kid is like a long journey for us. It is not only the distance but also the availability of the transport facility.* (PR – 4, F,46).

These factors had an inverse impact on vaccination coverage, resulting in an increased dropout rate and a higher number of children becoming defaulters.

#### Effects of culture on a child’s vaccination

During the in-depth interviews, participants shared their perspectives on how cultural norms impact vaccination coverage. Cultural norms affect both acceptance and refusal of vaccination. Many participants elaborated on how our cultural norms hinder the process of taking healthy children for vaccination.

*Our tradition tells us to treat children after illness, but why should I take them to health facilities? Allowing them to receive a vaccine injection without any illness could harm their health. Hence, I would never risk making my child sick by vaccinating them without any disease.* (PR – 9, M, 35).

#### Impact of religious beliefs

Religious considerations emerged as a major theme during the in-depth interviews.

Participants discussed how religious factors affect the completion of the recommended vaccination schedule for children. Religious beliefs significantly influence individuals’ perceptions, attitudes, and behaviours towards adhering to the vaccination schedule.

Decisions about completing a child’s vaccinations are often guided by religious leaders.

While in some cases, religious leaders played a positive role in encouraging vaccination, in most instances, their influence was negative. Participants mentioned that fatwas (religious decrees) were issued against vaccination, stating that vaccinating a child or adult is contrary to religious teachings.

*If our religious leader supports the vaccination programme, I will definitely complete my child’s vaccination schedule without any hesitation. However, since he does not approve of children’s vaccinations, I will not vaccinate my children anymore.* (PR –12, M, 59).

Furthermore, some religious rituals conflicted with the vaccination schedule, leading to dropouts from the recommended vaccination schedule.

#### Migration-related challenges

One of the themes that emerged during the in-depth interviews was the challenges related to migration. Families relocating from conflict areas face numerous obstacles in getting their children vaccinated. Participants explained that being constantly on the move makes it extremely difficult to manage children’s vaccinations. Some participants mentioned losing the vaccination card during their relocation, which led to the discontinuation of their child’s vaccination schedule.

*We recently moved to this area, and the transition was challenging for us. Everything here is unfamiliar, and we don’t know where to go to vaccinate our child. Additionally, it’s uncertain whether the vaccinator will agree to vaccinate our child since we are new to this area.* (PR –16, F, 55).

## Discussion

The objective of this study was to assess vaccination defaults among children under 2 years in Kohat District, Pakistan, and to explore sociodemographic and healthcare-related factors associated with incomplete immunization.

The quantitative finding in our study revealed that children younger than 4 months were significantly more likely to default on early vaccines such as OPV1, ROTA1, and PENTA1, consistent with national and regional evidence [23–24]. This highlights the extreme vulnerability of infants in their first months of life and emphasizes the need for targeted interventions during this critical window. From qualitative insights, the predominance of fathers (96.05%) among respondents reflects cultural norms in Khyber Pakhtunkhwa, where men commonly act as healthcare decision-makers. This respondent profile showed a potential bias, as mothers who are more directly involved in child health may provide distinct perspectives on barriers to immunization.

This study emphasizes the importance of age, showing that children younger than 4 months old are more likely to miss necessary immunizations such as OPV1, ROTA1, and PENTA1. According to earlier research, many Pakistani toddlers between the ages of 12 and 24 months do not adhere to recommended immunization schedules [25]. The most recent and comprehensive study on routine immunization, the ‘Third Party Verification Immunization Survey (TPVICS)’, conducted in Pakistan, also revealed that 5.9% of children under the age of two had never received any vaccinations, and more than one in six had only received partial vaccinations [26]. This highlights how vulnerable newborns are to missing out on vital immunizations during their first 4 months of life. One possible explanation for this significant correlation could be the difficulties & significant obstacles that caregivers had to overcome at this crucial time. Newborns frequently need more frequent doctor visits and vaccination appointments, which adds to the load for parents or guardians and may cause noncompliance. The observed tendency may also be explained by logistical problems, a lack of knowledge, or parental worries about the safety of immunizations for extremely young newborns [27].

It is important to notice both consistencies and divergences between our findings and those found in the literature on vaccine defaulter rates among home-born and institutionally born babies. According to a study conducted in India by Schön M et al., women who received more postnatal care had a higher percentage of vaccine compliance, but infants born in public or private settings were not more likely than those born at home to have received all recommended vaccinations [28]. On the other hand, our results are consistent with a study by Mekuria DK et al. that found mothers who gave birth at home are linked to their children’s failure to comply with immunization schedules [29]. This suggests that those who are born at home could encounter difficulties or obstacles when trying to follow the suggested immunization schedule. Those born at home, as opposed to those born in medical facilities, may have trouble getting timely and well-organized immunization services. A disorganized healthcare environment may be a factor in mistakes made regarding vaccination records, scheduling, or awareness.

The bulk of the non-adherent youngsters are migrants from other cities, which further demonstrates the statistically substantial impact of resident status and migration on compliance, according to our results. This result is consistent with other studies that have shown how susceptible immigrant groups are to vaccination defaults [30]. Due to issues like restricted access to healthcare services, unfamiliarity with the local healthcare system, and possible disruptions in routine healthcare practices during the migration process, studies in similar contexts have frequently reported higher default rates among migrant populations [31]. Our findings support these general patterns, but given the subtle variations in default rates, they also highlight the necessity of context-specific treatments.

According to Bugavi et al., one factor influencing Pakistani children’s inadequate immunization is the father’s kind of work [32]. We also discovered a similar pattern, wherein father unemployment and working abroad were linked to increased rates of vaccine default. Research conducted in a variety of contexts has regularly connected parental occupation to vaccine default, typically identifying time constraints, work-related restrictions, and financial issues as relevant variables [33]. Our findings support the existing relationships but also highlight the necessity for customized interventions given the particular situations involving fathers without jobs and those who work abroad. Possible reasons for the higher default rates among unemployed fathers may include family life disruptions, socioeconomic difficulties, or other stressors affecting healthcare priorities. On the other hand, the observed tendency may be attributed to various factors, including geographic separation, restricted access to healthcare information, and logistical challenges in arranging vaccination regimens across borders.

The significant correlation between vaccine default rates and non-mobile phone usage is consistent with previous research, which frequently emphasizes the importance of communication channels in healthcare adherence [34]. Research has indicated that those without access to a cell phone could find it difficult to get appointment notices, reminders, and educational messages in a timely manner, which increases the risk that they would miss vaccination appointments. Our findings highlight the significance of investigating alternative communication tactics to close the communication gap for those without mobile phones, such as community outreach initiatives or the use of other accessible technology.

Our findings of characteristics connected to healthcare providers are consistent with larger conversations about the influence of proximity on healthcare utilization in the literature [35]. As has been seen in a number of healthcare settings, vaccine adherence may be positively impacted by the ease of getting medical services within a 5-km radius. This result raises the possibility that initiatives aimed at placing mobile units or immunization clinics in close proximity to communities could be a useful tactic for lowering default rates.

Our study has a few limitations that need to be considered. First, because of budgetary limitations, we only admitted kids from one Pakistani district. Including kids from other regions might have improved our findings and allowed us to draw broader generalizations. Secondly, we were unable to verify whether the children who were not eligible for immunizations because of their age had received those specific vaccines.

Furthermore, it can be challenging to establish causal relationships in these kinds of studies between vaccine defaulters and independent variables. However, the results are helpful in directing future studies and efforts to raise immunization rates in the area.

This study has several limitations that should be considered when interpreting the findings. First, the cross-sectional design precludes establishing causal relationships between the identified determinants and immunization outcomes; longitudinal studies are needed to confirm temporal associations and assess intervention impacts. Second, while providing detailed insights into immunization barriers in Kohat District, the findings may not be fully generalizable to other regions of Pakistan due to variations in healthcare infrastructure, cultural practices, and vaccine confidence. Future multi-district or nationally representative studies are recommended to enhance external validity. Third, vaccination status was primarily based on caregiver report, which is susceptible to recall bias. However, this concern is substantially mitigated by the fact that 97.89% of participants provided verified immunization cards for record confirmation. Finally, as noted in the methodology, the precision of estimates for certain subgroups (e.g., specific maternal education categories) is limited due to small sample sizes within those strata, as reflected in the wide confidence intervals in our regression analyses. This affects the stability of point estimates for these particular variables but does not invalidate the primary findings related to core exposures like travel time and vaccine awareness. Our study had a high proportion of father respondents (96.05%), which may reflect cultural norms in Khyber Pakhtunkhwa where male family members often serve as primary decision-makers for healthcare. This could introduce bias, as mothers typically more involved in child healthcare may have differing perspectives on immunization barriers. More studies should actively engage mothers to capture a more comprehensive understanding of vaccination decision-making dynamics. Although the questionnaire was adapted from previously validated instruments and pretested in a pilot study to ensure clarity, flow, and cultural appropriateness, a full psychometric validation was not undertaken due to time and resource constraints. Future research could strengthen the tool’s reliability and construct validity through formal validation procedures in similar populations.

The qualitative findings complemented the quantitative results by providing contextual explanations for observed statistical patterns. For instance, while quantitative analysis identified child age, father occupation, and migration status as predictors of immunization default, the qualitative interviews revealed that limited maternal involvement, logistical challenges, and cultural perceptions of vaccine safety contributed to these patterns. Together, these findings highlight how structural and sociocultural factors jointly shape immunization behaviour.

## Conclusion

The findings indicate that vaccination default is influenced by a combination of socioeconomic, geographic, and knowledge-related factors. Parental education, particularly maternal education, was associated with improved immunization adherence, while children living in rural and peri-urban areas were more likely to experience vaccination default due to logistical barriers such as longer travel distances and extended travel time to healthcare facilities.

Caregiver knowledge also played an important role in vaccination behaviour. Although general awareness about vaccination was high, gaps persisted in understanding the immunization schedule and VPDs. The qualitative findings further highlighted contextual barriers such as accessibility challenges, migration, and sociocultural perceptions that influence adherence to the vaccination schedule. Together, these results suggest that both structural and knowledge-related determinants shape childhood immunization uptake.

### Recommendations

Efforts to improve immunization rates should focus on enhancing maternal education, addressing geographic accessibility in underserved areas, and promoting targeted community outreach to bridge knowledge gaps. These interventions have the potential to reduce default rates and improve vaccination coverage across vulnerable populations.

## Supporting information

10.1017/S0950268826101277.sm001 Ashfaq et al. supplementary material Ashfaq et al. supplementary material

## Data Availability

All the data generated and analysed in this study are included in this article.

## Supplementary material

The supplementary material for this article can be found at http://doi.org/10.1017/S0950268826101277.
