# Supplementary material for: Barriers and facilitators of childhood immunization: sociodemographic and knowledge-related determinants among parents in rural and urban Khyber Pakhtunkhwa, Pakistan
Source: Epidemiol Infect. 2026 Mar 23;154:e41. doi: 10.1017/S0950268826101277 (PMC13100931; doi:10.1017/S0950268826101277)
Supplement: Ashfaq et al. supplementary material [file S0950268826101277sup001.docx]

| **Supplementary Table S1: Descriptive Statistics** | |
| --- | --- |
| **Characteristic** | **n= 380**^1^ |
| ***Children Demographic***  **Age in months** | |
| less than 4 | 281 (73.95%) |
| 4-8 | 0 (0.00%) |
| 8.1-12 | 83 (21.84%) |
| more than 12 | 16 (4.21%) |
| **Gender** |  |
| Male | 230 (60.53%) |
| Female | 150 (39.47%) |
| ***Respondent characteristics***  **Residency** | |
| Urban | 131 (34.47%) |
| Urban Rural fringe area | 90 (23.68%) |
| Rural | 159 (41.84%) |
| **Terrain** |  |
| Mountain | 8 (2.11%) |
| Hill | 23 (6.05%) |
| Plain | 349 (91.84%) |
| **Maternal Education** |  |
| Illiterate | 13 (3.42%) |
| Primary | 98 (25.79%) |
| secondary | 48 (12.63%) |
| High School | 181 (47.63%) |
| College & Above | 40 (10.53%) |
| **Paternal Education** |  |
| Illiterate | 3 (0.79%) |
| Primary | 9 (2.37%) |
| secondary | 91 (23.95%) |
| High School | 110 (28.95) |
| College & Above | 167 (43.95%) |
| **Use of mobile** |  |
| No | 15 (3.95%) |
| Yes | 365 (96.05%) |
| ***Children characteristics***  **Ethnicity** | |
| Muslim | 379 (99.74) |
| Non muslim | 1 (0.26%) |
| **Birth place** |  |
| Hospital | 218 (57.37%) |
| Health Center | 153 (40.26%) |
| Home | 9 (2.37%) |
| **Resident Status** |  |
| Permeant resident | 359 (94.47%) |
| Migrant From another City | 6 (1.58%) |
| Migrant From another Province | 15 (3.95%) |
| ***Healthcare provider characteristics***  **Immunization provider** | |
| DHQ | 175 (46.05%) |
| BHU/RHC | 121 (31.84%) |
| Civil dispensary | 74 (19.47%) |
| Administration of vaccines at home | 10 (2.63%) |
| **Distance from home** |  |
| <5km | 79 (20.79%) |
| >5km | 231 (60.79%) |
| >10km | 70 (18.42%) |
| **Travelling time** |  |
| <20minutes | 76 (20.00%) |
| 21-40 minutes | 237 (62.37%) |
| >40minutes | 67 (17.63%) |
| **Respondent knowledge** | |
| Have you ever heard about vaccination | 362 (95.26%) |
| **No of VPD Known to you** |  |
| known one | 15 (3.95%) |
| known two | 40 (10.53%) |
| known three | 126 (33.16%) |
| Known four | 151 (39.74%) |
| Known Five or more | 46 (12.11%) |
| I don’t Know | 2 (0.53%) |
| **Age at which child start EPI services** |  |
| just after birth | 120 (31.58%) |
| at six weeks | 188 (49.47%) |
| any time | 52 (13.68%) |
| after one year | 13 (3.42%) |
| don’t Know | 7 (1.84%) |
| **No of sessions required to complete EPI** |  |
| One | 11 (2.89%) |
| Two | 108 (28.42%) |
| Three | 171 (45.00%) |
| Four | 73 (19.21%) |
| Five | 16 (4.21%) |
| Six | 1 (0.26%) |
| **Age at which child completes EPI** |  |
| 3 to 6 months | 144 (37.89%) |
| 6 to 8 months | 111 (29.21%) |
| 9 to 15 months | 125 (32.89%) |
| **Objectives of vaccination** |  |
| To prevent disease | 63 (16.58%) |
| For healthy child | 192 (50.53%) |
| It has no benefit | 104 (27.37%) |
| I don’t know | 21 (5.53%) |
| **Source of information** |  |
| Radio | 6 (1.58%) |
| School | 23 (6.05%) |
| health worker | 227 (59.74%) |
| Friend | 38 (10.00%) |
| Television | 65 (17.11%) |
| Others | 21 (5.53%) |
| **Availability of immunization book or card of Immunization** |  |
| No | 8 (2.11%) |
| Yes | 372 (97.89%) |
| **Defaulter for group of antigens** |  |
| OPV1, ROTA1 & PENTA1 | 20 (5.26%) |
| OPV2,ROTA2, PENTA 2 | 126 (33.16%) |
| OPV3,IPV 1 & Penta3 | 134 (35.26%) |
| MR1 | 84 (22.11%) |
| MR2 | 16 (4.21%) |
| ^1^n (%) | |

| **Supplementary Table 2: Sociodemographic determinants of defaulter parents for different vaccine antigens** | | | | | | |
| --- | --- | --- | --- | --- | --- | --- |
| **Characteristic** | **OPV1, ROTA1 & PENTA1** | **OPV2,ROTA2, PENTA 2** | **OPV3,IPV 1 & Penta3** | **MR1** | **MR2** | **p-value**^2^ |
|  | N = 20^1^ | N = 126^1^ | N = 134^1^ | N = 84^1^ | N = 16^1^ |  |
| **Respondent relationship** |  |  |  |  |  | 0.85 |
| Mother | 0 (0%) | 3 (2.4%) | 3 (2.2%) | 0 (0%) | 0 (0%) |  |
| Father | 20 (100%) | 119 (94%) | 128 (96%) | 82 (98%) | 16 (100%) |  |
| Grandparents | 0 (0%) | 4 (3.2%) | 3 (2.2%) | 2 (2.4%) | 0 (0%) |  |
| **Father occupation** |  |  |  |  |  | 0.082 |
| Jobless | 8 (40%) | 23 (18%) | 30 (22%) | 17 (20%) | 6 (38%) |  |
| Govt Servant | 4 (20%) | 22 (17%) | 28 (21%) | 26 (31%) | 1 (6.3%) |  |
| Labor | 6 (30%) | 37 (29%) | 26 (19%) | 13 (15%) | 3 (19%) |  |
| Out of Country Job | 0 (0%) | 23 (18%) | 30 (22%) | 12 (14%) | 3 (19%) |  |
| Businessman | 2 (10%) | 21 (17%) | 20 (15%) | 16 (19%) | 3 (19%) |  |
| **Residency** |  |  |  |  |  | <0.001 |
| Urban | 14 (70%) | 38 (30%) | 47 (35%) | 31 (37%) | 1 (6.3%) |  |
| Urban Rural fringe area | 6 (30%) | 33 (26%) | 32 (24%) | 18 (21%) | 1 (6.3%) |  |
| Rural | 0 (0%) | 55 (44%) | 55 (41%) | 35 (42%) | 14 (88%) |  |
| **Terrain** |  |  |  |  |  | 0.019 |
| Mountain | 0 (0%) | 0 (0%) | 3 (2.2%) | 3 (3.6%) | 2 (13%) |  |
| Hill | 1 (5.0%) | 6 (4.8%) | 7 (5.2%) | 6 (7.1%) | 3 (19%) |  |
| Plain | 19 (95%) | 120 (95%) | 124 (93%) | 75 (89%) | 11 (69%) |  |
| **Maternal Education** |  |  |  |  |  | 0.014 |
| Illiterate | 1 (5.0%) | 7 (5.6%) | 1 (0.7%) | 3 (3.6%) | 1 (6.3%) |  |
| Primary | 4 (20%) | 27 (21%) | 29 (22%) | 27 (32%) | 11 (69%) |  |
| secondary | 1 (5.0%) | 16 (13%) | 17 (13%) | 13 (15%) | 1 (6.3%) |  |
| High School | 13 (65%) | 62 (49%) | 71 (53%) | 32 (38%) | 3 (19%) |  |
| College & Above | 1 (5.0%) | 14 (11%) | 16 (12%) | 9 (11%) | 0 (0%) |  |
| **Paternal Education** |  |  |  |  |  | 0.001 |
| Illiterate | 1 (5.0%) | 2 (1.6%) | 0 (0%) | 0 (0%) | 0 (0%) |  |
| Primary | 1 (5.0%) | 3 (2.4%) | 1 (0.7%) | 4 (4.8%) | 0 (0%) |  |
| secondary | 2 (10%) | 27 (21%) | 27 (20%) | 24 (29%) | 11 (69%) |  |
| High School | 3 (15%) | 42 (33%) | 42 (31%) | 21 (25%) | 2 (13%) |  |
| College & Above | 13 (65%) | 52 (41%) | 64 (48%) | 35 (42%) | 3 (19%) |  |
| **Use of mobile** |  |  |  |  |  | 0.001 |
| No | 4 (20%) | 6 (4.8%) | 5 (3.7%) | 0 (0%) | 0 (0%) |  |
| yes | 16 (80%) | 120(95%) | 129 (96%) | 84 (100%) | 16 (100%) |  |
| **Child Gender** |  |  |  |  |  | 0.44 |
| Male | 13 (65%) | 73 (58%) | 77 (57%) | 58 (69%) | 9 (56%) |  |
| Female | 7 (35%) | 53 (42%) | 57 (43%) | 26 (31%) | 7 (44%) |  |
| **Ethnicity** |  |  |  |  |  | 0.001 |
| Muslim | 19 (95%) | 126 (100%) | 134 (100%) | 84 (100%) | 16 (100%) |  |
| Non muslim | 1 (5.0%) | 0 (0%) | 0 (0%) | 0 (0%) | 0 (0%) |  |
| **Birth place** |  |  |  |  |  | 0.001 |
| Hospital | 13 (65%) | 77 (61%) | 76 (57%) | 48 (57%) | 4 (25%) |  |
| Health Center | 4 (20%) | 46 (37%) | 56 (42%) | 35 (42%) | 12 (75%) |  |
| Home | 3 (15%) | 3 (2.4%) | 2 (1.5%) | 1 (1.2%) | 0 (0%) |  |
| **Resident** |  |  |  |  |  | 0.018 |
| Permanent resident | 18 (90%) | 119 (94%) | 127 (95%) | 79 (94%) | 16 (100%) |  |
| Migrant From another City | 2 (10%) | 4 (3.2%) | 0 (0%) | 0 (0%) | 0 (0%) |  |
| Migrant From another Province | 0 (0%) | 3 (2.4%) | 7 (5.2%) | 5 (6.0%) | 0 (0%) |  |
| **Immunization provider** |  |  |  |  |  | 0.047 |
| DHQ | 10 (50%) | 63 (50%) | 70 (52%) | 28 (33%) | 4 (25%) |  |
| BHU/RHC | 6 (30%) | 41 (33%) | 33 (25%) | 33 (39%) | 8 (50%) |  |
| Civil dispensary | 3 (15%) | 22 (17%) | 24 (18%) | 21 (25%) | 4 (25%) |  |
| Administration of vaccines at home | 1 (5.0%) | 0 (0%) | 7 (5.2%) | 2 (2.4%) | 0 (0%) |  |
| **Distance from home** |  |  |  |  |  | 0.052 |
| <5km | 8 (40%) | 28 (22%) | 30 (22%) | 13 (15%) | 0 (0%) |  |
| 5km – 10km | 8 (40%) | 81 (64%) | 78 (58%) | 54 (64%) | 10 (63%) |  |
| >10km | 4 (20%) | 17 (13%) | 26 (19%) | 17 (20%) | 6 (38%) |  |
| **Travelling time** |  |  |  |  |  | 0.24 |
| <20minutes | 7 (35%) | 27 (21%) | 26 (19%) | 16 (19%) | 0 (0%) |  |
| 21-40 minutes | 10 (50%) | 80 (63%) | 84 (63%) | 53 (63%) | 10 (63%) |  |
| >40minutes | 3 (15%) | 19 (15%) | 24 (18%) | 15 (18%) | 6 (38%) |  |
| **Availability of immunization book or card** |  |  |  |  |  | <0.001 |
| No | 3 (15%) | 2 (1.6%) | 1 (0.7%) | 1 (1.2%) | 1(6.3%) |  |
| Yes | 17 (85%) | 124 (98%) | 133 (99%) | 83 (99%) | 15 (94%) |  |
| **Age in months** |  |  |  |  |  | <0.001 |
| Less than 4 months | 20 (100%) | 126 (100%) | 134 (100%) | 0 (0%) | 0 (0%) |  |
| 4-8 months | 0 (0%) | 0 (0%) | 0 (0%) | 0 (0%) | 0 (0%) |  |
| 8.1-12 months | 0 (0%) | 0 (0%) | 0 (0%) | 84 (100%) | 0 (0%) |  |
| More than 12 months | 0 (0%) | 0 (0%) | 0 (0%) | 0 (0%) | 16 (100%) |  |
| **Gender** |  |  |  |  |  | 0.44 |
| Male | 13 (65%) | 73 (58%) | 77 (57%) | 58 (69%) | 9 (56%) |  |
| Female | 7 (35%) | 53 (42%) | 57 (43%) | 26 (31%) | 7 (44%) |  |
| **Maternal Education** |  |  |  |  |  | 0.014 |
| Illiterate | 1 (5.0%) | 7 (5.6%) | 1 (0.7%) | 3 (3.6%) | 1 (6.3%) |  |
| Primary | 4 (20%) | 27 (21%) | 29 (22%) | 27 (32%) | 11 (69%) |  |
| secondary | 1 (5.0%) | 16 (13%) | 17 (13%) | 13 (15%) | 1 (6.3%) |  |
| High School | 13 (65%) | 62 (49%) | 71 (53%) | 32 (38%) | 3 (19%) |  |
| College & Above | 1 (5.0%) | 14 (11%) | 16 (12%) | 9 (11%) | 0 (0%) |  |
| ^1^n / N (%) | | | | | | |
| ^2^Pearson's Chi-squared test/Fisher exact test | | | | | | |
